# Quality indicators for primary care and patients’ experience: A leap beyond

**DOI:** 10.1002/jgf2.319

**Published:** 2020-04-10

**Authors:** Luiz Felipe Pinto, Erno Harzheim, Otávio Pereira D’Avila, Lisiane Hauser

**Affiliations:** ^1^ Department of Primary Health Care in Medicine School of Medicine Federal University of Rio de Janeiro (UFRJ) Rio de Janeiro Brazil; ^2^ Instituto de Higiente e Medicina Tropical (IHMT) Universidade Nova de Lisboa Nova de Lisboa Portugal; ^3^ School of Medicine Federal University of Rio Grande do Sul (UFRGS) Rio de Janeiro Brazil; ^4^ School of Dentist Federal University of Pelotas, Rio Grande do Sul (UFPel) Rio de Janeiro Brazil; ^5^ Brazilian Health Ministry Rio de Janeiro Brazil

## 1 THE IMPORTANCE OF PATIENTS’ EXPERIENCE IN ASSESSING THE QUALITY OF PRIMARY CARE

Ozaki et al (2019)^1^ had evaluated the quality of primary care throughout small facilities using quality indicators (QIs) collected in electronic medical records (EMR) system. They selected 18 QIs based on a comprehensive framework after a modified Delphi method validated by an expert panel members. Besides, the authors also collected data on a patient survey in four dimensions presented in the Appendix: access, communication, coordination, and understanding of patient's background. But, unfortunately, these dimensions are not comparable to other studies in primary health facilities. What do we mean by that? We ask why not use a statistically validated international instrument to assess patients' experience in primary care clinics?

To measure quality of Japan's medical care, one should argue what patients’ experience is. They play an important role in the assessment of health services, especially in primary care, where clinics and facilities are usually near each person/family. Starfield and Shi mentioned by the authors on references #1 and #4 had developed and validated a set of questionnaires to evaluate primary healthcare facilities—the Primary Care Assessment Tool (PCAT),^2^ and recently, Aoki et al^3^ also validated a Japanese adult version, the so‐called J‐PCAT. It has most of the dimensions considered by the authors, which together allow the measurement of an overall IQ (the overall PCAT score), in addition to allowing the specific calculation for each of the attributes: access—first contact, ongoing care, comprehensiveness (services available), comprehensiveness (services provided), and community orientation.

## 2 THE USE OF PCAT‐BRAZIL AND THE NATIONAL HEALTH SURVEY (PNS‐2019): A LEAP BEYOND

The use of PCAT is widespread in health services in Brazil, in the states of São Paulo, Rio de Janeiro and especially in Rio Grande do Sul. In agreement with Ozaki et al (2019),^1^ when they mention that primary care plays an *“increasingly important role in the healthcare system*,” we propose a leap beyond. Why not outline a national policy for primary care assessment including users’ perspective? Thinking about this issue, in early 2019, the Brazilian Ministry of Health sought Brazilian Institute of Geography and Statistics (IBGE)—the Statistics Bureau of Brazil—to include in its main National Health Survey (PNS‐2019), the set of questions that forms Brazilian Adult PCAT reduced version.^4^, ^5^

Therefore, we encourage the authors in future studies to focus on expanding their research in community clinics in Japan using J‐PCAT for the assessment of patients’ experience, helping to foster a baseline to compare nationwide primary care facilities. It could be used not only for health service research, but for a Japanese primary care policy, as Brazilian Federal Government is innovating since 2019, considering it for Family Health Teams (that form the basis of the health system in Brazilian primary care), to evaluate the quality of primary care throughout a part of a pay‐for‐performance program implemented.

## CONFLICT OF INTEREST

The authors declare no conflict of interests for this article.
